# Serum lipophilic antioxidants levels are associated with leucocyte telomere length among US adults

**DOI:** 10.1186/s12944-018-0781-x

**Published:** 2018-07-20

**Authors:** Mohsen Mazidi, Andre Pascal Kengne, L. J. Cheskin, Maciej Banach

**Affiliations:** 1 Department of Biology and Biological Engineering, Food and Nutrition Science, Chalmers University of Technology, SE-412 96 Gothenburg, Sweden; 2 Non-Communicable Disease Research Unit, South African Medical Research Council and University of Cape Town, Cape Town, South Africa; 3 Department of Health, Johns Hopkins Weight Management Center Behavior and Society, Johns Hopkins Bloomberg School of Public Health, Johns Hopkins University, Baltimore, MD USA; 4 Global Obesity Prevention Center at Johns Hopkins University, International Health/Human Nutrition, Johns Hopkins University Bloomberg School of Public Health, Baltimore, MD USA; 5 Department of Hypertension, Chair of Nephrology and Hypertension, Medical University of Lodz, Lodz, Poland; 6 Polish Mother’s Memorial Hospital Research Institute (PMMHRI), Lodz, Poland; 7 Cardiovascular Research Center, University of Zielona-Gora, Zielona Gora, Poland

**Keywords:** Telomere length, Antioxidant, National health and nutrition examination survey

## Abstract

**Background:**

To examine the association between serum concentrations of antioxidant and telomere length (TL) in U.S adults.

**Methods:**

Participants of the National Health and Nutrition Examination Survey (NHANES) with data available on TL measures from 2001 to 2002 were included. Serum lipophilic antioxidants level was measured using high performance liquid chromatography with photodiode array detection. We used analysis of co-variance and multivariable-adjusted linear regression models, accounting for the survey design and sample weights.

**Results:**

Of the 5992 eligible participants, 47.5% (*n* = 2844) were men. The mean age was 46.9 years overall, 47.2 years in men and 46.6 in women (*p* = 0.071). In age, sex, race, education, marital status, adiposity, smoking, C-reactive protein adjusted linear regressions, antioxidant, serum α-carotene, trans-β-carotene, cis- β-carotene, β-cryptoxanthin and combined Lutein/zeaxanthin were positively and significantly associated with TL (all *p* < 0.001).

**Conclusions:**

Our findings support a possible positive association between serum concentrations of lipophylic antioxidant and TL. The implications of this association deserve further investigation.

## Background

Telomeres are repetitive DNA sequences bound by specialized interacting proteins that are found at the ends of chromosomes [1]. Telomeres serve multiple functions: They maintain chromosome stability and integrity, regulate cellular proliferation, and prevent chromosome end fusions [2]. Telomeres are progressively eroded due to successive rounds of cell division, and due to such processes as oxidative stress [3]. Endogenous (i.e., genetics, inflammation, and DNA damage) and environmental (i.e., smoking, alcohol, and life stress) factors are involved in telomere maintenance and regulation [4–7]. Therefore, variation in telomere length (TL) between individuals of equal chronological age, could be due to differences in both genetic factors and environmental determinants (including adiposity) [4–7].

Shorter leukocyte TL is associated with aging [8, 9] and age-related diseases, such as cardiac disease [10, 11], diabetes [12, 13], hypertension [12, 14, 15], cancer [16], as well as with increased mortality [17]. Shorter LT is associated with greater oxidative stress [18, 19]. In this regard, evidence from previous in vitro, in vivo [19], and clinical studies [10, 12, 20] suggests that an imbalance between free oxygen radicals and antioxidant concentration in the cellular environment contributes to telomere attrition. Epidemiological studies report an inverse association between dietary micronutrients and oxidative stress [21], which suggests that dietary micronutrients may exert protective effects on TL through anti-oxidative effects. Antioxidants have attracted attention as an efficient tool in counteracting oxidative stress [22]. A limited number of studies have investigated the association between self-reported dietary antioxidants and TL [23, 24], and only a handful of studies have evaluated the relationship between TL and objectively measured serum levels of antioxidants; with however, inconsistent findings. A study examined the association between the plasma levels of antioxidative micronutrients and the leukocyte telomere length in 786 elderly adults, who had participated in the Austrian Stroke Prevention Study and reported a significantly protective effect of combined lutein/zeaxanthin on the telomere length [25]. There is still insufficient evidence regarding the link between serum antioxidant and TL in subjects.

The aim of this study was to investigate the relationship between serum antioxidant levels with TL among US adults. We have hypothesized that subjects with higher level of the anti-oxidants have longer length of telomere.

## Methods

### Population

The National Health and Nutrition Examination Surveys (NHANES) are ongoing, repeated cross-sectional surveys conducted by the US National Center for Health Statistics (NCHS). NHANES uses a multistage probability sampling strategy, which oversamples certain subgroups of the population, including blacks, Mexican-Americans, and those of lower socioeconomic status. The NCHS Research Ethics Review Board approved the NHANES protocol and consent was obtained from all participants. About 5000 subjects participate in NHANES each year, and the data are reported in 2-year cycles available for public use. Data collection on demographic, dietary, and behavioural information occurs through home interview-administered questionnaires, while anthropometric and biomarker data are collected by trained staff using mobile exam units. The interviews consist of questions on socio-demographic characteristics (age, gender, education, race/origin, and health insurance) and questions on previously diagnosed medical conditions. More details on the NHANES protocol is available elsewhere [26]. Details on the measurement of C-reactive protein (CRP) concentrations are available elsewhere [27]. This study was based on analysis of data from the 2001–2002 NHANES cycle. Subject less than 18 years, pregnant and lactating were excluded.

For the assessment of height and weight during the physical examination, participants were dressed in underwear, disposable paper gowns and foam slippers. A digital scale was used to measure weight to the nearest 100 g, a fixed stadiometer was used to measure height to the nearest millimetre. Body mass index (BMI) was calculated as weight in kilograms divided by the square of height in metres [27]. Smoking status was self-reported and participants classified as current smoker or not.

### Leucocyte telomere measurements

Aliquots of purified DNA, isolated from whole blood using the Puregene (D-50 K) kit protocol (Gentra Systems, Inc., Minneapolis, Minnesota), were obtained from participants. The leukocyte TL assay was performed using the quantitative polymerase chain reaction method to measure TL relative to standard reference DNA (also known as the [telomere-to–single-copy gene ratio (T/S) ratio) [28]. Of note, this method provides an average TL from leukocytes (92 telomeres for humans). It does not have the capabilities to detect specific individual TL. Laboratory personnel were blinded to all other study measurements. The Centers for Diseases Control (CDC) conducted a quality control review before linking the TL data to the NHANES data files. The CDC Institutional Review Board granted human subject approval for this study [29].

### Antioxidants measurements

A blood specimen was drawn from the participant’s antecubital vein by a trained phlebotomist. Serum concentrations of retinol, α and γ-tocopherol, two retinyl esters, and six carotenoids (α-carotene, trans-β-carotene, cis β-carotene, β-cryptoxanthin, combined lutein/zeaxanthin, and trans-lycopene) were measured using high performance liquid chromatography with photodiode array detection [26]. Serum β -carotene concentrations predominantly reflect *trans* β -carotene, which was present in substantially higher concentrations than cis β -carotene. The CVs for these micronutrients were 1.9–5.7% for retinol, 1.8–3.9% for α -tocopherol, 1.9–6.2% for γ -tocopherol, 4.2–18.3% for α -carotene, 3.5–6.6% for trans β -carotene, 7.5–65% for cis β -carotene, 3.0–7.1% for β -cryptoxanthin, 5.5–13.4% for lutein + zeaxanthin, and 3.7–13.1% for lycopene [26].

### Statistical analyses

We conducted analyses in accordance with CDC guidelines for analysis of complex NHANES data, accounting for the masked variance and using the proposed weighting methodology [30]. We have calculated adjusted (age, gender and sex) mean of the serum anti-oxidant level across the TL quarters by analysis of co-variance (ANCOVA). To determine any association between antioxidants and TL, we used both crude and multivariable-adjusted (age and sex and race and education and marital status and BMI and smoking and CRP) linear regression models. All tests were two sided, and *p* < 0.05 was the level of significance. Data were analysed using SPSS complex sample module version 22.0 (IBM Corp, Armonk, NY). Sample weights were applied to account for unequal probabilities of selection, nonresponse bias, and oversampling.

## Results

Of the 5992 eligible participants, 47.5% were men. The mean age was 46.9 years overall, 47.2 years in men and 46.6 in women (*p* = 0.071). With regard to education, 45.5% had completed more than high school, 23.4% had completed high school, while 30.8% had completed less than high school. Whites (non-Hispanic) represented 50.6% of the participants, blacks 19.8% and Mexican-Americans 21.7%. With regard to marital status, 51.4% were married, 10.2%were widowed and 7.6% divorced.

Table 1 shows the sex-age-adjusted mean of serum antioxidant levels across quartiles of TL. Adjusted mean of α-carotene, *trans*-β-carotene, *cis*-β-carotene, β-cryptoxanthin, combined lutein/zeaxanthin, *trans*-lycopene, and retinol levels increased as the length of telomere increased (all *p* < 0.001). Moreover there was an inverse association between γ-tocopherol and TL in crude model, but after adjustment for potential confounders (age and sex and race and education and marital status and BMI and smoking and CRP) the association between γ-tocopherol and TL was no longer significant. In multiple linear regressions, α-carotene, *trans*-β-carotene, *cis*-β-carotene, β-cryptoxanthin, and combined lutein/zeaxanthin remained positively associated with TL (all *p* < 0.001) (Table 2).

**Table 1:** Age and sex adjusted mean of serum antioxidant across quartiles of telomere length

Variables	Quarters of telomere length				
	Q1	Q2	Q3	Q4	P-trend
*N*	1068	1057	1068	1066	
*TL, Mean ± SEM*	0.76 ± 0.02	0.95 ± 0.01	1.10 ± 0.04	1.41 ± 0.10	
α-carotene(umol/L)	0.062 ± .004	0.080 ± 002	0.084 ± 008	0.087 ± 003	< 0.001
trans-β-carotene(umol/L)	0.29 ± .012	0.35 ± .01	0.36 ± .01	0.38 ± .01	< 0.001
cis- β -carotene(umol/L)	0.017 ± .001	0.020 ± .001	0.021 ± .001	022 ± .001	< 0.001
β -cryptoxanthin(umol/L)	0.172 ± .005	0.197 ± .005	0.196 ± .005	0.195 ± .005	< 0.001
g-tocopherol(umol/L) *(vitamins E)*	5.93 ± .10	5.75 ± .10	5.66 ± .10	5.30 ± .10	< 0.001
Combined Lutein/zeaxanthin(umol/L)	0.270 ± .005	0.289 ± .005	0.295 ± .005	0.297 ± .005	< 0.001
trans-lycopene(umol/L)	0.398 ± .006	0.411 ± .006	0.417 ± .006	0.417 ± .006	< 0.001
Retinyl palmitate(umol/L)	0.085 ± .003	0.081 ± .003	0.086 ± .003	0.094 ± .003	< 0.001
Retinyl stearate(umol/L)	0.018 ± .001	0.016 ± .001	0.017 ± .001	0.019 ± .001	< 0.001
Retinol(umol/L) *(vitamins A)*	2.08 ± .020	2.09 ± .018	2.11 ± .018	2.11 ± .018	< 0.001
α -tocopherol(umol/L) *(vitamins E)*	30.88 ± .46	31.39 ± .43	31.77 ± .43	31.12 ± .45	< 0.001

*P*-values for linear trend across quartiles of telomere length. Variables were compared across quartiles of telomere length using analysis of co-variance (ANCOVA) test

**Table 2:** Crude and multi-variable (age-sex-race-education-*marital status*-BMI-Smoking-CRP) association between telomere length and serum antioxidant

Variables	Crude model		Multivariable model	
	β	95% CI	β	*p*-value
α-carotene(umol/L)	0.021	(− 0.019,0.113)	0.058	(0.246, 0.807)
trans-β-carotene(umol/L)	−0.026	(− 0.037,0.003)	0.060	(0.081,0.251)
cis- β -carotene(umol/L)	−0.026	(−0.663, 0.051)	0.061	(1.52,4.59)
β -cryptoxanthin(umol/L)	0.041	(0.016,0.108)	0.049	(0.112,0.501)
g-tocopherol(umol/L)	−0.047	(−0.006,-0.001)	− 0.016	(− 0.062,0.126)
Combined Lutein/zeaxanthin(umol/L)	− 0.023	(− 0.082, 0.010)	0.400	(0.062,0.495)
trans-lycopene(umol/L)	0.142	(0.132, 0.203)	0.017	(−0.066,0.238)
Retinyl palmitate(umol/L)	−0.002	(−0.087,0.074)	0.015	(−0.178,0.498)
Retinyl stearate(umol/L)	−0.052	(−0.714, − 0.187)	0.004	(− 0.959,1.261)
Retinol(umol/L)	− 0.035	(− 0.062,0.040)	0.006	(− 0.039,0.058)
α -tocopherol(umol/L)	− 0.050	(− 0.516,0.0129)	0.008	(− 0.002,0.003)

Linear regression, adjusted for age-sex-race-education-marital status-BMI-Smoking-CRP

## Discussion

We investigated the association between serum antioxidants and TL among US adults. We have found a positive association between serum levels of α-carotene, *trans*-β-carotene, *cis*-β-carotene, β–cryptoxanthin and combined lutein/zeaxanthin with TL.

Our findings were in line with other studies that have suggested a positive association with dietary and supplemental α-tocopherol intake [23–25, 31]. Consistent with our findings, two previous studies observed longer TL in participants with higher self-reported dietary β-carotene intake [23–25, 31]. However, for γ-tocopherol, our data suggested no association, which is in line with the previous study among 786 Austrian adults [23–25, 31].

The performance of vitamin A and its pro-vitamin (e.g. β and α-carotene) has rarely been evaluated in relation to TL. Vitamin A has widespread physiologic roles, including in immune function, vision, reproduction, and cell communication. Among the plausible mechanisms by which vitamin A and its pro-vitamin could influence TL are its roles in immune function, inflammation, and the regulation of gene expression and epigenetic modifications [32, 33]. Two previous studies assessed the associations between serum vitamin A and TL [24, 25]. We observed a significant positive association between TL with serum β and α -carotene. This is consistent with the previously reported positive association between dietary intake of vitamin A and TL in a study of 2284 women [24]. Conversely, plasma vitamin A concentrations were not associated with LTL in a study of 786 adults [25]. Carotenoids are particularly promising antioxidants, because they are capable of exerting antioxidant protection by scavenging singlet molecular oxygen and peroxyl radicals [34]. Several human studies have supported carotenoid protection against oxidation and oxidative stress-induced, age-related conditions [34]. We hypothesized that protective role of vitamin A and its procures could be at least partially related to its antioxidant activity.

Also consistent with our findings, the Austrian Stroke Prevention Study (ASPS) investigated the association of longer TL with higher plasma lutein, zeaxanthin, and vitamin C concentrations [25]. Findings of this study indicated that higher lutein and zeaxanthin concentrations in serum were related to longer TL, though they found that no other antioxidants (nor the pooled subgroups of provitamin A, non-provitamin A, vitamin E, and total antioxidant status of all micronutrients) were associated with TL. The authors proposed a protective role of these specific vitamins in telomere maintenance [25]. Further, the European Prospective Investigation into Cancer and Nutrition (EPIC) found that a higher consumption of vegetables was meaningfully associated with higher mean TL in peripheral lymphocytes. Particularly, β-carotene consumption significantly increased mean TL [35]. A recent study by Min, et al. from Japan reported for the first time a positive association between TL and daily intake of antioxidant vitamins, particularly among certain genotypes [31, 35]. Sen et al. investigated the association between plasma antioxidant levels and leukocyte telomere length in 786 elderly adults. A significant effect of combined lutein/zeaxanthin on longer telomeres was observed, whereas no other carotenoids were associated with telomere length [25].

Several potential mechanisms have been proposed for a positive association between antioxidant levels and TL [25, 36–38]. It is consistent with our findings that antioxidants would maintain TL [31, 39]. Oxidative stress is considered to be an important contributing factor for telomere shortening because of the high guanine content in telomeres. Oxidative stress is considered to be an important contributing factor for telomere shortening because of the high guanine content in telomeres. Guanine is highly sensitive to reactive oxygen species, resulting in the production of 8-oxo-7,8-dihydrodeoxyguanosine, which can lead to DNA strand breaks, resulting in telomeric attrition [1]. Antioxidant vitamins, including a-tocopherol, g-tocopherol, and carotenoids, may limit the effects of oxidative stress and possibly slow telomeric attrition. Several in vitro and in vivo studies have shown that lutein, zeaxanthin and vitamin C prevent DNA breakage and modulate DNA repair through their anti-oxidative activity [25, 36–38]. Moreover, previous clinical investigations proposed that non-pro-vitamin A carotenoids have greater protective effects against DNA damage than pro-vitamin A carotenoids [25, 36]. Other proposed mechanisms, particularly for the protective effect of lutein, zeaxanthin and vitamin C, include effects on immune-modulation, anti-inflammatory activity, modulation of apoptosis, and lymphocyte proliferation [25, 40, 41]. Several investigations conducted on other dietary components, including B vitamins like folate [42–44] point to their role in DNA methylation and consequently regulation of TL by DNA methylation [43].

Even though we have adjusted our models for potential confounders, our results should be considered with caution as it has been reported higher serum antioxidant levels with higher intakes of vegetables and fruits, and also with higher socioeconomic status (SES) [45]. Therefore, the positive association between certain dietary antioxidants and TL may also be explained by a more healthy life style/higher SES. It should also be realized that oxidative stress as it occurs with chronic inflammation may cause consumption of certain serum antioxidants [46] as well as a reduction of TL, thereby contributing to the association between certain antioxidants and TL. The cross-sectional nature of our study does not allow inferences about causality. Circulating levels of nutrients may be a marker for an overall healthier lifestyle. Although we accounted for several lifestyle factors, such as BMI, smoking, and physical activity level, it is possible that the inclusion of these covariates did not completely account for factors that may confound the associations between vitamins and TL. In the current study we just focused on Leucocytes TL, more studies on other TL are warranted. The strength of the study could be its large sample size and the high quality of the methods measuring antioxidants and TL. The NHANES is a large nationally representative sample of non-institutionalized adults with comprehensive biomarkers and health information. An additional strength is the use of objectively measured biomarkers in the analysis rather than relying on self-reported dietary intake.

## Conclusion

In conclusion, results of the present study support the potential role of dietary antioxidants in preserving TL. This potential protective role of high antioxidant foods on TL has clinical implications with respect to biological aging and non-communicable disease. Further longitudinal studies are needed to confirm our findings, and to test whether changes in dietary antioxidants would preserve TL over time.

## Abbreviations

ANCOVA: Analysis of co-variance
ASPS: Austrian stroke prevention study
BMI: Body mass index
CDC: Centers for diseases control
CRP: C-reactive protein
EPIC: European Prospective Investigation into Cancer and Nutrition
NCHS: National Center for Health Statistics
NHANES: National health and nutrition examination survey
TL: Telomere length
